# Drivers of phenological changes in southern Europe

**DOI:** 10.1007/s00484-022-02331-0

**Published:** 2022-07-26

**Authors:** Johannes Vogel

**Affiliations:** 1 Theoretical Ecology, Institute of Biology, Freie Universität Berlin, Königin-Luise-Straße 2/4, 14195 Berlin, Germany; 2 Institute of Environmental Science and Geography, University of Potsdam, Karl-Liebknecht-Str. 24-25, 14476 Potsdam, Germany

**Keywords:** Phenology, Southern Europe, Plant functional types, Linear mixed effect model, Climate change

## Abstract

The life cycle of plants is largely determined by climate, which renders phenological responses to climate change a highly suitable bioindicator of climate change. Yet, it remains unclear, which are the key drivers of phenological patterns at certain life stages. Furthermore, the varying responses of species belonging to different plant functional types are not fully understood. In this study, the role of temperature and precipitation as environmental drivers of phenological changes in southern Europe is assessed. The trends of the phenophases leaf unfolding, flowering, fruiting, and senescence are quantified, and the corresponding main environmental drivers are identified. A clear trend towards an earlier onset of leaf unfolding, flowering, and fruiting is detected, while there is no clear pattern for senescence. In general, the advancement of leaf unfolding, flowering and fruiting is smaller for deciduous broadleaf trees in comparison to deciduous shrubs and crops. Many broadleaf trees are photoperiod-sensitive; therefore, their comparatively small phenological advancements are likely the effect of photoperiod counterbalancing the impact of increasing temperatures. While temperature is identified as the main driver of phenological changes, precipitation also plays a crucial role in determining the onset of leaf unfolding and flowering. Phenological phases advance under dry conditions, which can be linked to the lack of transpirational cooling leading to rising temperatures, which subsequently accelerate plant growth.

## Introduction 

Phenological shifts serve as a prominent and long-term proxy of climate change with the oldest observations dating back to the ninth century (Aono and Kazui 2008; Menzel 2002; Menzel et al. 2020). It is well established from observational and experimental studies that phenological cycles are altered by climate change, e.g., leaf unfolding and flowering have been advancing by approximately 5–6 days per °C in recent decades (Piao et al. 2019; Wolkovich et al. 2012). These shifts in the onset of phenological phases influence a large variety of environmental and ecological processes. Phenology determines the length of the growing season and plays therefore a substantial role in the annual water, nutrient and carbon cycles (Forrest and Miller-Rushing 2010; Wang et al. 2022). Climate change alters temperatures, whereas photoperiod remains unchanged. Therefore, species-specific phenological responses to climate change potentially differ, which can cause decoupling of interactions between species. These mismatches can have detrimental consequences for ecosystem functioning and plant fitness (Anderson 2016; Hoegh-Guldberg et al. 2018). Furthermore, climate change alters the phenology of pests with potentially adverse impacts on crop yields, e.g., by accelerating the life cycle of pathogenic fungi (Lamichhane et al. 2015; Luck et al. 2011).

In southern Europe, the growing season is mainly constrained by low temperatures in winter and water limitation in summer, leading to a bimodal cycle of ecosystem productivity with two peaks — a larger one in spring and a smaller one in autumn — with a high seasonal variability of carbon uptake (Camarero et al. 2021; Garbulsky et al. 2008; Gutiérrez et al. 2011; Spano et al. 2013). This region is termed a global change hot spot, where warming rates exceed the global average by about 20% (Diffenbaugh and Giorgi 2012; Lionello and Scarascia 2018). In combination with increasing dryness, this has potentially detrimental effects on ecosystems (Gordo and Sanz 2009; Samaniego et al. 2018). Southern Europe has a broad variety of plant functional types with varying phenological responses to environmental drivers (Gordo and Sanz 2009; Richardson et al. 2013), which has only received limited attention so far. Furthermore, due to the comparatively small amount of available data, there is less research on phenology in southern Europe compared to central Europe (Cook et al. 2012b; García-Mozo et al. 2010). For example, the effects of precipitation on phenological variability in water-limited regions have not been addressed in depth so far (Mazer et al. 2015). This highlights the suitability of southern Europe as a study area for the systematic assessment of the influence of meteorological drivers on the onset of phenological phases.

The timing of phenological events is driven by complex interactions between organisms and environmental factors, whose individual importance is often not fully known (Forrest and Miller-Rushing 2010; Parmesan and Hanley 2015). For example, the role of vernalization and the importance of photoperiod in comparison to temperature as an environmental cue for wild plants are not well understood (Flynn and Wolkovich 2018; Parmesan and Hanley 2015). Furthermore, the influence of precipitation on phenological phases is ambiguous, depending highly on phase, species, and timing (Menzel et al. 2011; Spano et al. 2013; Wolkovich et al. 2012). Drought can, e.g., lead to earlier flowering by increasing temperatures via reduced evaporative cooling, but it can also delay phenophases by inhibiting plant growth (Bernal et al. 2011; Spano et al. 2013). Therefore, a joint investigation of precipitation and temperature effects at various time steps preceding phenological events is crucial to disentangle their effects on phenological trends and to gain a better understanding of the underlying mechanisms. In the following, this study addresses two main questions: (a) how did phenological phases shift for different plant functional types and (b) which are the main climatological drivers of phenological patterns in southern Europe within the time span 1951–2019?

## Materials and methods

### Data

The phenological data sets were retrieved from PEP725 (Templ et al. 2018), the phenological network of the Spanish Meteorological Agency (AEMET, https://sede.aemet.gob.es/AEMET/es/GestionPeticiones/home) and Tempo (https://data.pheno.fr), containing time series for the period from 1951 to 2019 south of 47°N latitude excluding Austria and Switzerland due to their alpine climate. The phenological dates are available in the BBCH scale (Biologische Bundesanstalt, Bundessortenamt, and CHemical industry (Meier et al. 2009; Meier 2018). In most cases, the altitude of the locations is given. In cases where information on altitude was not available, the altitude of the corresponding grid point of the European Digital Elevation Model (EU-DEM v1.1, https://land.copernicus.eu/imagery-in-situ/eu-dem) was taken (Meier 2018).

Daily mean temperature and precipitation were obtained from E-OBS. This data set provides data at a spatial resolution of 0.1° for Europe. Gordo and Sanz (2010) found that mean temperature has a higher explanatory power than maximum and minimum temperature for modeling phenological phases in Spain. Therefore, mean temperature was selected as a predictor variable in this study instead of maximum or minimum temperature.

### Data processing

**Fig. 1:**
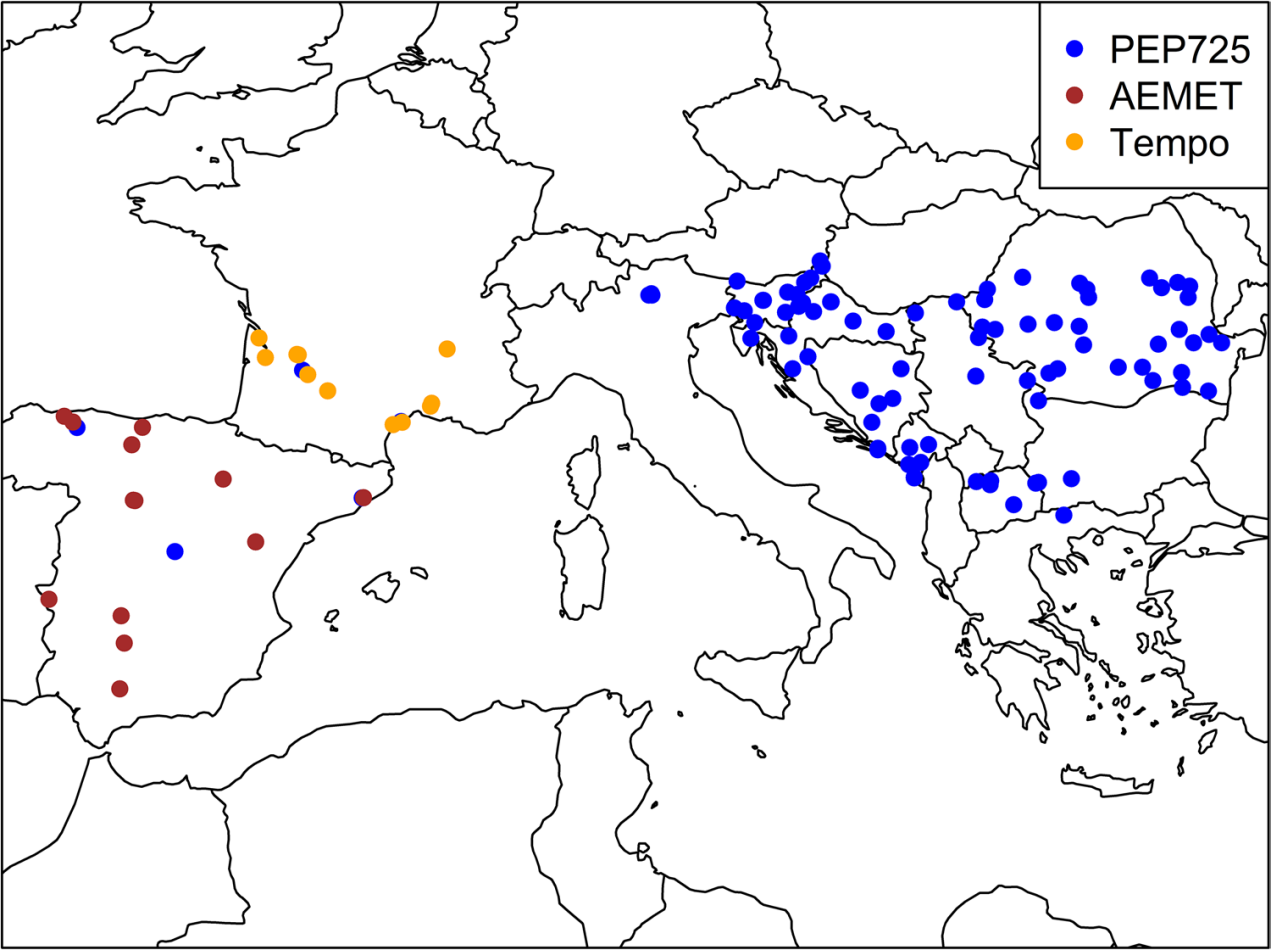
Locations of phenological time series of the PEP725 (blue), AEMET (red), and Tempo (orange) datasets

The dates of phenological observations were converted into the corresponding day of the year (DOY). In cases where more than one observation per year within a time series at a given location was available, the average of the respective DOYs was calculated (Gordo and Sanz 2010; Mazer et al. 2015; Menzel et al. 2020). Phenological observations of species with a remark on impairment by pests or diseases were excluded. Outliers beyond the threefold interquartile range were discarded, assuming that these entries are erroneous. The locations of all phenological time series are shown in Fig. 1. The phases from the BBCH scale were aggregated into four main phenophases (leaf unfolding, flowering, fruiting, senescence) according to Table 2. The species were assigned to the plant functional types (1) deciduous broadleaf tree, (2) evergreen needle leaf tree, (3) evergreen broadleaf tree, (4) deciduous shrub, (5) crop, and (6) perennial herb, similar to the procedure by Estrella et al. (2009).

A 30-day moving window was applied to calculate 30-day mean temperature *tg* and 30-day precipitation sum *rr*. For each time series, deviations from the mean phenological date *DOY*_*dev*_ were calculated. In addition, deviations from the 30-day mean temperature *tg*_*dev*_ and 30-day precipitation sum *tg*_*dev*_ were calculated by subtracting the corresponding long-term mean of the respective 30-day mean and sum, respectively. The climate data were then normalized using *z* score transformation to ensure comparability of the predictor coefficients (Templ et al. 2017). t*g*_*dev*_ and *rr*_*dev*_ for the time spans 0–29, 30–59, 60–89, 90–119, and 120–149 days prior to the DOY are used, hereafter referred to as l0, l30, l60, l90, and l120. Finally, each phenological time series was linked to the climatological time series of the respective grid point of its geographic coordinates in the E-OBS data set.

Many studies link all phenological observations of a given time series to an average date, either corresponding to the average date of the respective phenophase or even just corresponding to the respective average calendar month (Cook et al. 2012b). However, this leads to a certain misalignment of climate and phenological data, since the actual date of a given year deviates from the average date. This can lead to unrealistic cases of mismatches, where e.g., the phenological date of a given year is prior to the corresponding date of the climatological data, and thus could not have been influenced by it. Therefore, a more flexible approach was used in this study, where each phenological observation was linked with the respective climatological entry at the same date instead of a fixed singular date. This leads to higher temporal precision, while at the same time the importance of growing degree days over time is still incorporated since a 30-day running mean and sum is applied to the temperature and precipitation data, respectively. Furthermore, using deviations from mean climatological conditions instead of absolute temperature and precipitation values, a potential bias from temperature and precipitation seasonality is avoided.

We performed two types of linear regression analyses. First, a regression of *DOY*_*dev*_ against time for assessing the trend of phenophases. Only time series with a length of at least 30 years were included to ensure a time span of sufficient length for trend detection. Second, a regression of *DOY*_*dev*_ against climatological predictors to investigate their impact on phenological patterns for individual species (see Eq. (1)) as well as jointly for all species (see Eq. (2)):

1 $${\mathrm{DOY}}_{\mathrm{dev}}=\mathrm{tg}_{\mathrm{dev}}^{\mathrm{l0}}\ast\mathrm{rr}_{\mathrm{dev}}^{\mathrm{l0}}+\dots+\mathrm{tg}_{\mathrm{dev}}^{\mathrm l120}\ast\mathrm{rr}_{\mathrm{dev}}^{\mathrm l120}+\mathrm{lon}+\mathrm{lat}+\mathrm{alt}$$

2 $${\mathrm{DOY}}_{\mathrm{dev}}=\mathrm{tg}_{\mathrm{dev}}^{\mathrm{l0}}\ast\mathrm{rr}_{\mathrm{dev}}^{\mathrm{l0}}+\dots+\mathrm{tg}_{\mathrm{dev}}^{\mathrm l120}\ast\mathrm{rr}_{\mathrm{dev}}^{\mathrm l120}+\mathrm{lon}+\mathrm{lat}+\mathrm{alt}+(1\vert\mathrm{species})$$

For each combination of species and phenological phase, a linear model was calculated using the deviations of mean temperature *tg*_*dev*_ and precipitation sum *rr*_*dev*_ and their interaction at the time lags l0, l30, l60, l90, and l120 as well as latitude *lat*, longitude *lon*, and altitude *alt* as fixed effects to account for spatial variability (see Eq. (1)). Furthermore, we also calculated a linear mixed effects model for each phenophase (Wolkovich et al. 2012) including all corresponding species as random effect indicated by the (1|*species*) term (see Eq. (2)). For the climatological regression analysis, all time series of at least 15 years length were included.

A sensitivity analysis using several minimum time series lengths to assess the statistical robustness of the obtained results was carried out (Cook et al. 2012b; Menzel et al. 2020). All calculations were carried out for 15-, 20-, and 30- year minimum time span criteria. For the climatological regression, very similar results were obtained in all 3 cases. For this reason, it was decided to apply the least strict criterion of a 15-year minimum time span to minimize the loss of data. However, for the trend analysis, the number of statistical significant time series is reduced when applying shorter minimum time lengths (Menzel et al. 2020). Presumably, a 15- or 20-year time series lacks the sufficient length for performing a trend analysis, therefore the stricter 30-year minimum criterion is applied here. The number of time series, plant species, and locations is indicated in Table 3.

The statistical significance of each climatic predictor variable at each time lag was calculated for all combinations of phenophases and species. The *p* values were adjusted for multiple testing using the Benjamini and Hochberg (1995) correction. Note that for the linear mixed effect model, no *p* values were calculated, because these values are only approximate for linear mixed effect models and thus their informative value is limited (Zuur et al. 2009).

The analysis was carried out using R version 4.0 and Climate Data Operators (CDO) version 1.9 (R Core Team 2020; Schulzweida 2019). The linear mixed effects models were fitted using the lme4-package (Bates et al. 2015), and several figures were created using the ggplot2-package (Wickham 2016).

## Results

**Fig. 2:**
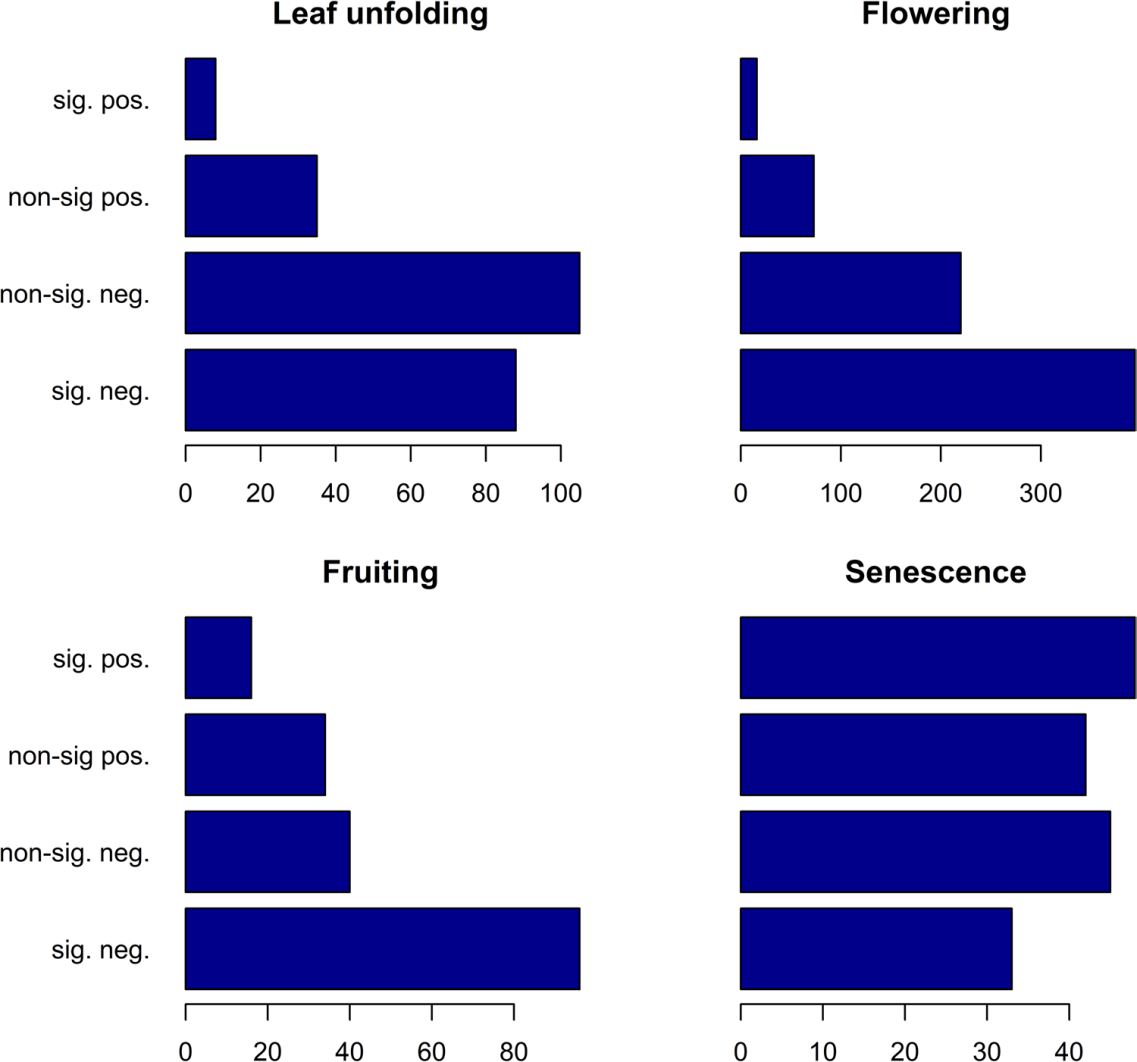
The number of (non-/)significantly positive and (non-/)significantly negative time series ranging within the time frame 1951–2019 for the 4 phenophases leaf unfolding, flowering, fruiting, and senescence

The vast majority of time series show a negative trend (Fig. 2 and Table 1) for the stages leaf unfolding (81.9%), flowering (87.4%), and fruiting (73.1%). For 37.4%, 56.0%, and 51.6% of all time series the trend is statistically significantly negative. Positive trends are much less frequent (18.0%, 12.6%, and 26.9%) and rarely statistically significant (3.4%, 2.3%, and 8.6%) for these three stages. The trend analysis for senescence does not reveal any clear pattern, as positive (53.3%) and negative trends (46.7%) are almost equally distributed.

**Table 1:** Proportion of negative, significantly negative, positive and significantly positive trends as well as the mean trend with standard error for each phenophase

Phenophase	Negative trends	Sig. negative trends	Positive trends	Sig. positive trends	Mean trends (days/year)
Leaf unfolding	0.82	0.37	0.18	0.03	− 0.123 ± 0.012
Flowering	0.87	0.56	0.13	0.02	− 0.186 ± 0.018
Fruiting	0.73	0.52	0.27	0.09	− 0.190 ± 0.059
Senescence	0.46	0.20	0.54	0.29	0.003 ± 0.016

**Fig. 3:**
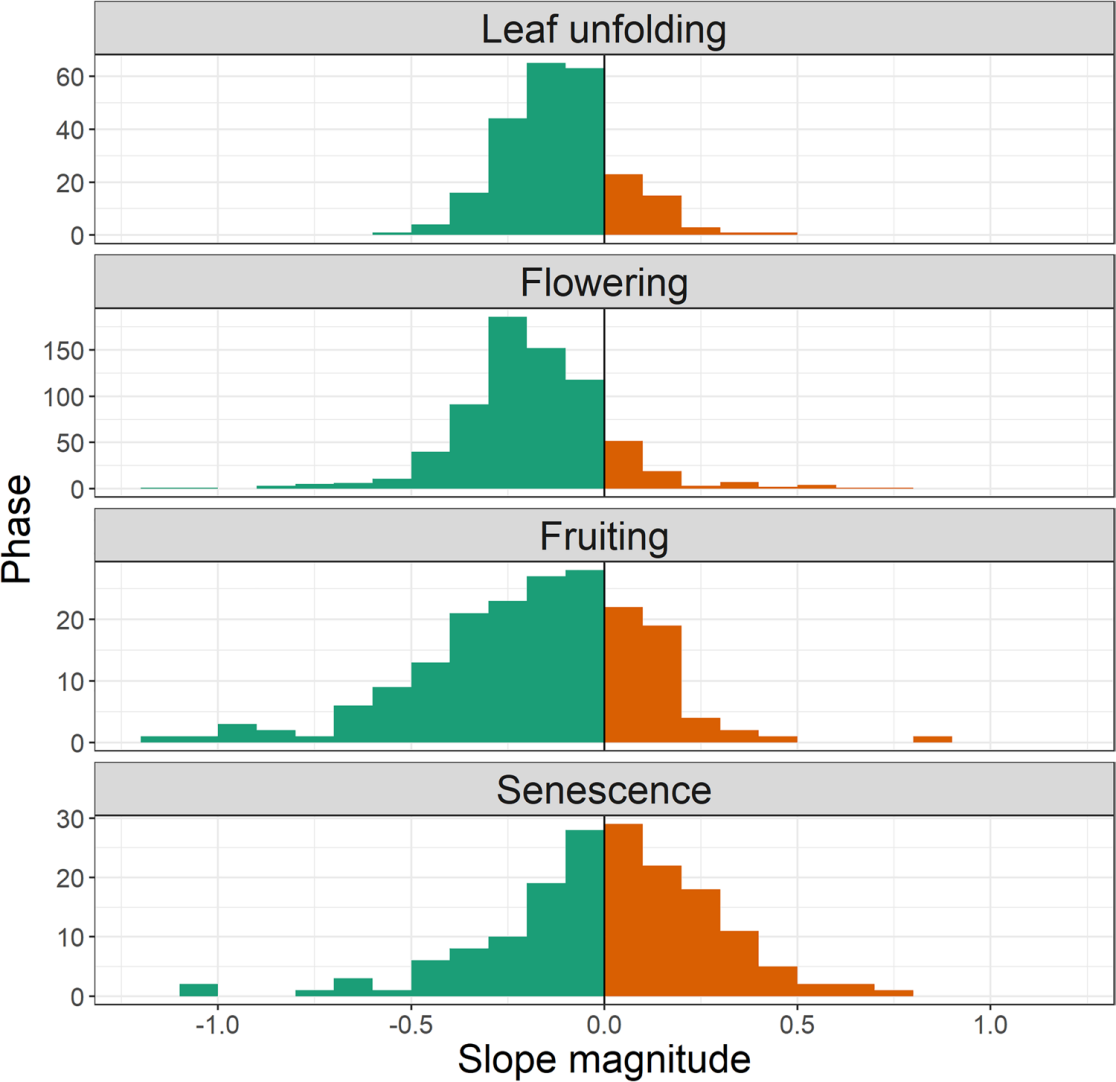
Magnitude of regression slope of phenological trends days/year for all species for leaf unfolding, flowering, fruiting, and senescence for all time series ranging within the time frame 1951–2019

The mean magnitude of the regression slope of all species is − 0.123, − 0.186, − 0.190, and 0.003 days/year for leaf unfolding, flowering, fruiting, and senescence, respectively (Fig. 3). The standard error of the regression slope is relatively small for leaf unfolding (0.012), flowering (0.018), and senescence (0.016) in comparison to fruiting (0.059) (Table 1). The small standard error for leaf unfolding and flowering indicates that most species show a similar behavior during these phases, whereas for fruiting and senescence the pattern is more diverse. The distribution of slope magnitudes in these late phenophases also has a relatively heavy tail compared to leaf unfolding and flowering.

We investigated the variance inflation factor (VIF) for each model to account for multicollinearity. The VIF did not exceed a value of 3 for any of the climatological predictors in the models, indicating that the level of collinearity between predictor variables is acceptable (Zuur et al. 2009).

**Fig. 4:**
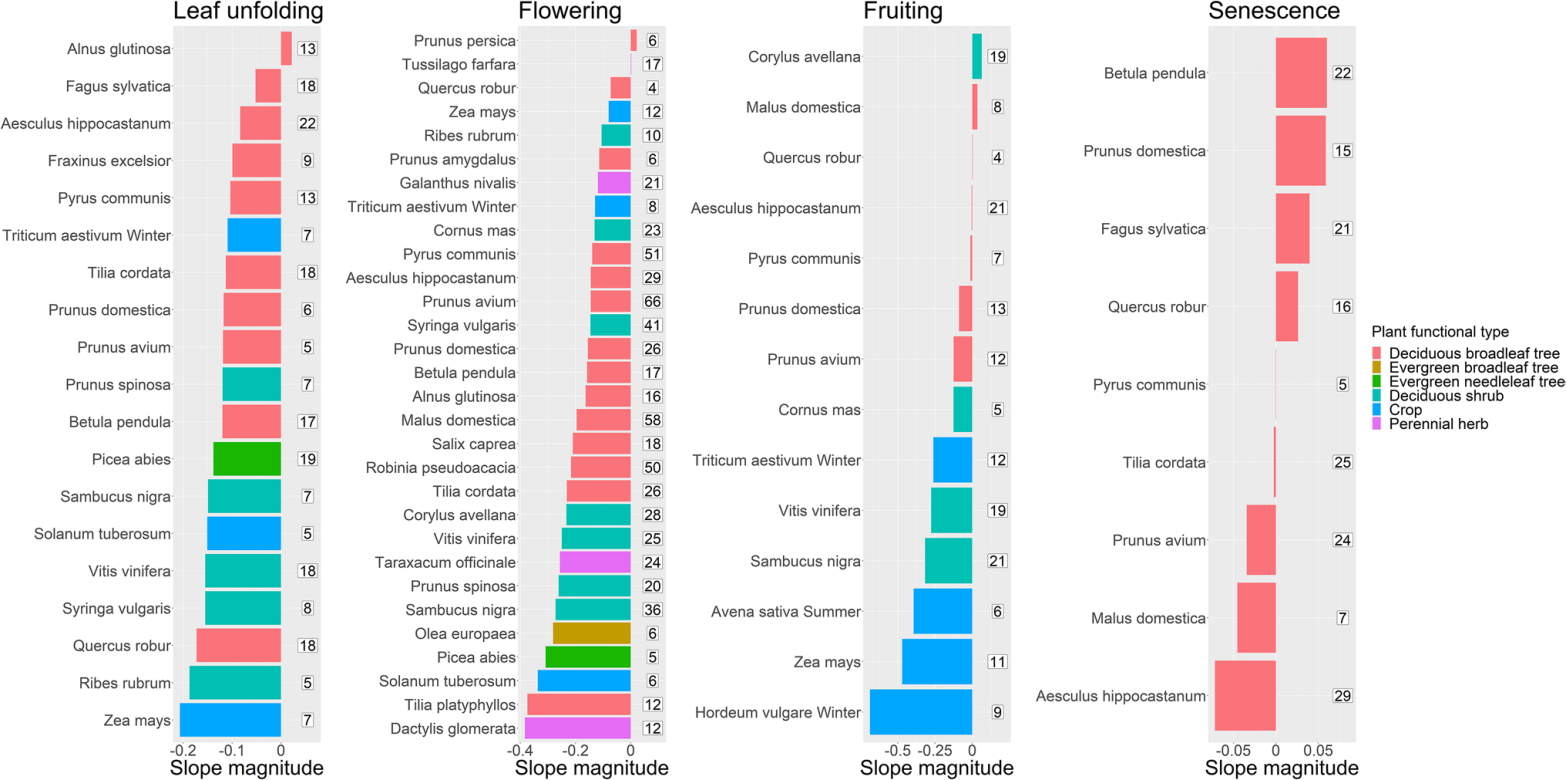
Mean magnitude of regression slope of phenological trends (days/year) per species and phenophase for all time series ranging within the time frame 1951–2019. The corresponding number of time series is stated on the right side of each bar. Only species with more than 3 time series available are shown. The color of the bar indicates the corresponding plant functional type. The addenda “Winter” and “Summer” indicate the season of cultivation for crops

Only for a few species the dates of leaf unfolding, flowering and fruiting shift to later DOYs (Fig. 4). Interestingly, the slopes of deciduous broadleaf trees are usually less pronounced than the slopes of deciduous shrubs during leaf unfolding and flowering. However, there are a few exceptions, e.g., *Quercus robur* during leaf unfolding and *Tilia platyphyllos* during flowering with magnitudes of − 0.173 and − 0.374, respectively. Also, fruiting dates are accelerating at faster rates for deciduous shrubs and crops with a median magnitude of − 0.201 and − 0.432 days/year compared to a median magnitude of − 0.008 days/year for deciduous broadleaf trees. The median slope magnitude is − 0.108 (− 0.157) days/year for deciduous broadleaf trees and − 0.155 (− 0.233) days/year for deciduous shrubs during leaf unfolding (flowering), respectively. Only deciduous broadleaf tree species are available during senescence, which makes it infeasible to compare the behavior of plant functional types during that phenophase.

**Fig. 5:**
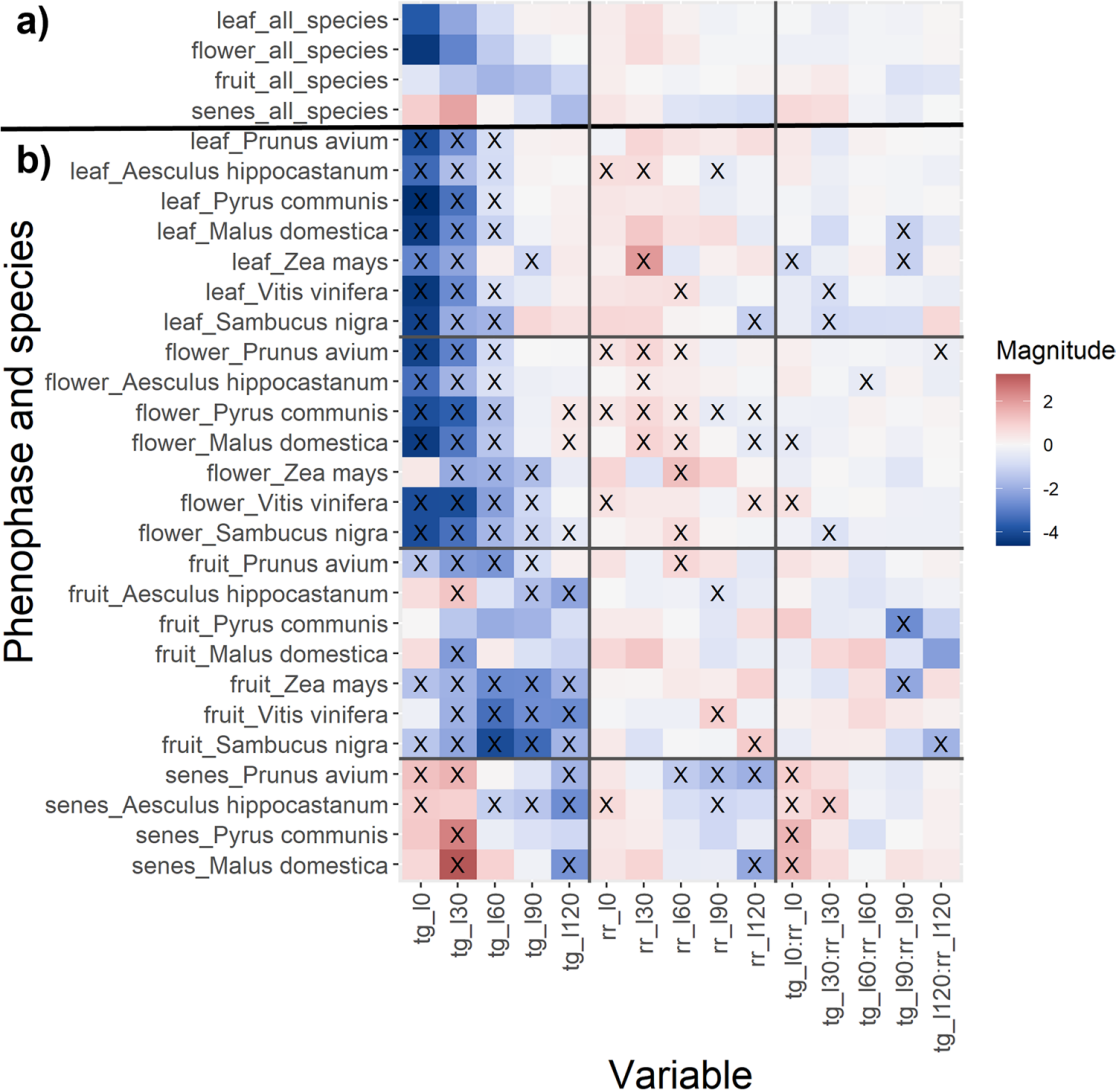
Regression coefficients of explanatory variables for the phenophases leaf unfolding, flowering, fruiting and senescence obtained from (**a**) a linear mixed effect model for all species and (**b**) linear models for a selection of exemplary species for time series ranging within the time frame 1951–2019. Mean temperature (tg_dev_) and precipitation sum (rr_dev_) deviations, as well as their interaction term (tg_dev_:rr_dev_) are shown for the time lags l0, l30, l60, l90, and l120. Coefficients significantly different from 0 are marked by black crosses. Note that no statistical significance is given for the linear mixed effects model for all species

Furthermore, the magnitude and statistical significance of climatic predictors were analyzed for all species (Fig. 5a) as well as a selection of exemplary species (Fig. 5b), where the species with a high number of time series for each phenophase were selected, including four broadleaf deciduous trees, two broadleaf shrubs and one crop species. Leaf unfolding, flowering, and fruiting are negatively correlated with temperature in most of the cases. This indicates that higher temperatures advance the occurrence of these phenophases, while lower temperatures delay them. The temperature of the 2–3 months prior to the phenological date mainly determines leaf unfolding and flowering. Flowering is also influenced by the temperature 4 months ago for some species such as *Zea mays*. For fruiting temperature in all 5 preceding months plays an important role, especially in the 2–4 months prior to the event. Senescence is positively correlated to temperature in the preceding 2 months, whereas it is negatively for months further back in time. However, temperature is often not significant as a predictor of the DOY of senescence.

Precipitation is positively correlated with the DOY of phenophases in the majority of cases. This shows that dry conditions usually lead to an earlier onset of phenophases, while wet conditions delay them. Precipitation is generally less often a significant predictor of the date of phenophases than temperature, and the magnitudes of precipitation coefficients are lower than the magnitudes of temperature coefficients. Nevertheless, precipitation is an important driver for certain species: for example, precipitation 2–3 months prior to the onset of flowering plays a significant role. Precipitation is seldom a significant predictor for leaf unfolding and fruiting. For senescence, significant negative correlation occurs for a few species, such as *Prunus avium*, 3–5 months prior to the event. Interaction terms of temperature and precipitation are not significant predictors of the DOY of phenophases in many cases. However, there are some notable exceptions, e.g., the importance of such interactions during the first month preceding senescence.

## Discussion

### Significance and magnitude of phenological trends

The proportion of negative, significantly negative, positive, and significantly positive trends is comparable to the respective values obtained by Menzel et al. (2020) for Europe. The magnitude of the mean phenological trend for the spring phases leaf unfolding and flowering (− 0.240 days/year) and for fruiting (− 0.256 days/year) in Europe according to Menzel et al. (2020) is higher compared to our findings, whereas the magnitude of the trend of senescence is negligible (0.036 days/year), in agreement to our findings. This indicates that phenological shifts in southern Europe are less pronounced compared to the average European shift. This is in line with findings by Templ et al. (2017), who state less pronounced advancements for Mediterranean and Pannonian areas compared to alpine and continental areas. However, it should be noted that substantial phenological changes also have been identified previously in southern Europe, e.g., for Spain by Gordo and Sanz (2009).

The advancement of the phenophases leaf unfolding, flowering, and fruiting of deciduous broadleaf trees is generally slower compared to other plant functional types. This deviates from previous findings identifying stronger advancements in the flowering of woody species compared to herbaceous species in studies by García-Mozo et al. (2010) and Templ et al. (2017). However, both studies only use a comparatively small number of species including only 7 and 6 species, respectively. Furthermore, it should be noted that the respective observations are primarily based on alpine and boreal regions in the study by Templ et al. (2017). In addition, the selected broadleaf tree species — *Robinia pseudoacacia* and *Tilia cordata* — might not be representative of deciduous broadleaf trees in general, as they advance faster than most other species of this plant functional type according to our findings, where they show the second and third strongest advancements in the flowering of all deciduous broadleaf trees, only superseded by *Tilia platyphyllos*.

### Variable importance

Temperature is regarded as the main driver of the onset of phenological phases (Cleland et al. 2007; Gordo and Sanz 2005, 2010). Increasing temperatures usually cause strong advances in spring during leaf unfolding and flowering, while advances are less pronounced during fruiting and a small delay of senescence occurs (Gordo and Sanz 2009; Menzel et al. 2020; Penuelas et al. 2002; Piao et al. 2019; Stuble et al. 2021). This is in line with our findings indicating a negative correlation between temperature and the onset of leaf unfolding, flowering and fruiting. While there is no clear pattern regarding senescence, a positive correlation prevails in the months prior to senescence. Additionally, the flexible approach applied here — which investigates the deviations of driver variables in the preceding 30-, 60-, 90-, 120, and 150-day time span of each phenological date — allows for an accurate identification of the most relevant time spans. This is more precise than the common approach in the scientific literature using fixed calendar months, which might lack the required precision in case phenophases do not coincide exactly with calendar months (Cook et al. 2012a; Seyfert 1960). The results indicate that temperature during the months directly prior to the onset phenological phases is particularly relevant for leaf unfolding and flowering, while for fruiting also the temperature 3–4 months ago is of equal importance.

In contrast to temperature, the influence of water availability on phenological dates is more elusive depending highly on phase, species, and timing (Jentsch et al. 2009; Menzel et al. 2011; Peñuelas et al. 2004; Wang et al. 2022; Wolkovich et al. 2012). Water availability has a lower influence on phenology in temperate regions in comparison to temperature (Piao et al. 2019) but is nevertheless a relevant predictor for Mediterranean phenology — where the onset of the rainy season is crucial — especially for autumn phenology (Luo et al. 2018; Matesanz et al. 2009; Peñuelas et al. 2004). Drought can influence phenological dates in two opposing ways. On the one hand, the lack of transpirational cooling increases leaf temperatures, which can accelerate plant growth, whereas on the other hand, reduced water availability constrains plant development (Bernal et al. 2011; Spano et al. 2013). Furthermore, dry conditions contribute to the earlier onset of leaf unfolding, presumably due to increased radiation (Wang et al. 2022). Therefore, the effects of changes in water availability differ strongly in the scientific literature, especially regarding the impact on flowering dates.

Both delays and accelerations of phenological dates have been recorded in southern Europe under dry conditions. For example, delayed watering led to a later onset of autumn flowering in an experimental study by Dios Miranda et al. (2009). Dry conditions also delayed the flowering of *Erica multiflora* and *Globularia alypum* — two common species of the Mediterranean coastal shrublands — in a field experiment by Llorens and Peñuelas (2005). However, in a follow-up study, the spring growing season of *Erica multiflora* advanced during experimental drought, while *Globularia alypum* showed no advances (Bernal et al. 2011). Furthermore, leaf unfolding and flowering in Spain advanced under warm and dry conditions according to findings by Gordo and Sanz (2010). However, the authors attribute this largely to temperature, rather than precipitation. In addition, Spano et al. (1999) found that drought advances flowering for non-native species in the Mediterranean, while native species were not affected. Finally, Peñuelas et al. (2004) noted that the effects of increased precipitation vary largely between species. While most species advanced leaf unfolding and flowering, some species did not exhibit significant changes and in case of *Vicia faba* even significant delays were found for flowering dates. Our results show that dry conditions predominantly delay phenological dates of leaf unfolding, flowering, and fruiting for the majority of plants in southern Europe. Only senescence advances sometimes under dry conditions. Most observations in this study are obtained from relatively mesic sites. Therefore, the observed accelerated phenological development under dry conditions is likely caused by a lack of evapotranspirational cooling, while the degree of drought severity might usually still be insufficient to substantially inhibit plant growth.

In a study by Flynn and Wolkovich (2018), neither temperature nor photoperiod could be clearly identified as the dominant cue of spring phenology of woody species in temperate forests. Nevertheless, long-lived, late-successional species, which encompasses most of the deciduous broadleaf tree species investigated here, are usually photoperiod-sensitive (Körner and Basler 2010). This sensitivity to photoperiod could counterbalance the influence of rising temperatures. This might explain why the advancement of leaf unfolding, flowering, and fruiting is less pronounced for those species in comparison to deciduous shrubs and crops in our findings. Crops such as *Avena* spp. are not sensitive to the photoperiod in Mediterranean regions (Loskutov 2001), and thus, their life cycle is mostly driven by temperature. Notably, in our findings, the summer breed of *Avena sativa* shows one of the highest advances in fruiting, together with other crop species including *Zea mays* and the winter breed of *Hordeum vulgare* (see Fig. 4). In future, the sensitivity to photoperiod could slow the advancing of phenophases despite rising temperatures for certain species (Körner and Basler 2010). This is in line with findings by Fu et al. (2015) and Menzel et al. (2020) who noted that the slopes of phenological trends are already slowing down in recent years. Furthermore, there is also evidence that the influence of minimum temperature was reduced at the expense of photoperiod and soil moisture over the last decades (Garonna et al. 2018).

Global warming particularly affects early phenophases, while the influence on later phases is less pronounced, sometimes even undetectable and generally a higher variance is observed for these phases (Piao et al. 2019; Spano et al. 2013; Sparks and Menzel 2002; Stuble et al. 2021). This can potentially be attributed to the interplay of several factors which play a role here (Gallinat et al. 2015). Late phenological phases vary depending on plant functional traits, and they do not only depend on the climatic conditions of the prior weeks but are additionally dependent on earlier phenophases and the complex interplay of environmental drivers during these phases (Bucher and Römermann 2021; Estiarte and Peñuelas 2015; Fu et al. 2014; Piao et al. 2019; Richardson et al. 2013). For example, warming can delay senescence, whereas summer drought — which often coincides with summer heat — can induce earlier senescence (Estiarte and Peñuelas 2015; Piao et al. 2019). Due to these counteracting effects, it remains unclear how the timing of senescence is changed under increasingly hot and dry conditions (Estiarte and Peñuelas 2015). The significant interaction term of precipitation and temperature in the 30 days preceding senescence in our findings clearly shows that the interplay of these drivers plays a major role in determining the date of this phenophase.

While high temperatures usually advance spring phenology, a warm winter can also lead to unfulfilled chilling requirements for some species and thus delay bud break (Fernández-Pascual et al. 2019; Murray et al. 1989). Species with insignificant trends or delays in flowering are likely more sensitive to photoperiod and/or reduced vernalization (Cook et al. 2012b; Fu et al. 2015). However, the role of vernalization in Mediterranean regions remains uncertain and varies between species (Spano et al. 2013). Meteorological changes can have further indirect effects, which might influence phenology and plant growth in the long term. For example, dry conditions lead to a reduction of phosphorus availability in evergreen Mediterranean forests, which adds further plant stress in addition to the water limitation (Sardans and Peñuelas 2004). In addition to climatic drivers, other environmental factors such as CO_2_ and nitrogen fluctuations also influence phenological responses (Cleland et al. 2006).

### Limitations

There is a higher focus on spring phenology in comparison to autumn phenology in data collection and research (Gallinat et al. 2015; Sparks and Menzel 2002). This is reflected in our data, which contains primarily information on spring phenophases, while there is a smaller number of species and time series per species available for fruiting and particularly senescence. For senescence, time series with a sufficient length are only available for deciduous broadleaf trees, which inhibits the comparison to other plant functional types for this phase. In general, data in southern Europe is relatively scarce in comparison to central Europe (Cook et al. 2012b; Estrella et al. 2009). Additionally, data availability varies largely between countries, with the majority of the data being provided by West Balkan countries. In summary, a more even representation of all major plant functional types for all phenological stages for a broader variety of countries would be highly desirable for future work to gain a better understanding of the differences between phenophases and plant functional types.

## Conclusion

A clear acceleration of the onset of leaf unfolding, flowering, and fruiting was found for almost every species. Interestingly, the acceleration is less pronounced in deciduous broadleaf trees compared to deciduous shrubs and crops, which could be explained by the higher constraint by photoperiod in case of deciduous broadleaf trees. While temperature in the 2–3 months preceding a phenological phase is clearly the major driver of phenological patterns, precipitation in the antecedent 1–2 months is also especially relevant for leaf unfolding and flowering. Whether precipitation (and the lack thereof) delays or advances phenophases is ambiguous in the scientific literature. Notably, the findings of this study indicate that there is usually a positive relationship between precipitation and the onset of phenophases for most species, i.e., wet conditions delay the onset, whereas dry conditions advance them.

Climatic extremes, such as the joint occurrence of heatwaves and droughts are especially increasing in spring in southern Europe (Vogel et al. 2021), which is the peak of the growing season (Schönfelder and Schönfelder 2018). The impact of such extreme events differs vastly between species and phenophases, but is expected to have substantial impacts on crop phenology if they occur during sensitive stages (Jentsch et al. 2009; Menzel et al. 2011; Moriondo and Bindi 2007). The investigation of the influence of increased frequency and severity of climatic extremes on phenology should therefore be addressed in future research. This highlights the need to account precisely for the seasonal timing of changes in temperature and precipitation (Forrest and Miller-Rushing 2010). However, many studies are confined to temperature and precipitation corresponding to calendar months or even annual means. The procedure presented in this article allows for a precise identification of the points in time where meteorological drivers have significant impacts on the phenological stages of plants. This setting is also easily transferable to other regions, and its flexibility can help to improve the understanding of processes at higher temporal resolution than usually applied in phenological studies. This is especially relevant for agricultural applications, where predictors based on calendar months can often only provide a limited understanding of a plant’s life cycle, which requires a more refined submonthly temporal scale.

## Appendix

Table 2

Table 3

**Table 2:** Aggregation of phenological phases

Aggregated phases	Phenological codes according to the BBCH scale (Meier 2018)^1^
Leaf unfolding	5, 7, 9, 10, 11, 12, 13, 14, 15, 31, 223
Flowering	50, 51, 53, 55, 59, 60, 61, 62, 63, 65, 67, 69
Fruiting	71, 75, 79, 80, 81, 83, 85, 87, 89, 286
Senescence	92, 93, 94, 95, 97, 201, 205, 209

^1^The phases 201, 205, 209, 223, and 286 are exclusive to the PEP725 data set (description available at http://www.pep725.eu/pep725_phase.php, last accessed on 28.06.2022) and are not covered in the BBCH scale.

**Table 3:** Metadata of phenological time series for various minimum time series lengths

Threshold for minimum length in years	Number of time series	Number of plant species	Number of locations
15	2335	81	114
20	1766	63	70
30	1293	44	45
